# The impact of new government childcare accreditation standards on children’s in-care physical activity and sedentary time

**DOI:** 10.1186/s12889-022-12888-5

**Published:** 2022-03-29

**Authors:** Valerie Carson, Zhiguang Zhang, Nicholas Kuzik, Kristi B. Adamo, Madison Predy, Mitchell Crozier, Stephen Hunter, Nancy Ogden, Gary S. Goldfield, Anthony D. Okely

**Affiliations:** 1 Faculty of Kinesiology, Sport, and Recreation, University of Alberta, Edmonton, AB Canada; 2 Faculty of Health Science, University of Ottawa, Ottawa, ON Canada; 3 Faculty of Arts, Mount Royal University, Calgary, AB Canada; 4 Children’s Hospital of Eastern Ontario Research Institute, Ottawa, ON Canada; 5 Early Start and Illawarra Health and Medical Research Institute, Faculty of Arts, Social Sciences and Humanities, University of Wollongong, Wollongong, NSW Australia

**Keywords:** Childcare, Policy, Movement behaviours

## Abstract

**Background:**

A new physical activity and sedentary behaviour accreditation standard criterion for childcare settings was introduced by the provincial government in Alberta, Canada. The primary objective of this study was to examine if changes for in-care physical activity and sedentary time (ST) differed between centres in and around Edmonton, Alberta after implementing the new accreditation standards and non-accredited control centres in and around Ottawa, Ontario. Secondary objectives were to examine whether baseline age group (toddler, preschooler) or the childcare environment moderated any group differences in change of the primary outcomes. Furthermore, accreditation and control group differences in change of children’s body mass index (BMI) Z-scores or cognitive development as well as educators’ perceptions of the primary outcomes were explored.

**Methods:**

Participants were 252 toddlers (19–35 months) and preschoolers (36–60 months) in childcare centres from Alberta (*n* = 11) and Ontario (*n* = 8) in the supporting Healthy physical AcTive CHildcare setting (HATCH) study. In-care ST, light-intensity physical activity (LPA), and moderate- to vigorous-intensity physical activity (MVPA) were accelerometer–derived before and 6 months after the implementation of the new standards. At both time points, cognitive development (working memory, expressive vocabulary), heights, and weights were measured, and BMI Z-scores were calculated. Additionally, the childcare environment was observed using the Environment and Policy Assessment and Observation (EPAO) and Movement Environment Rating Scale (MOVERS) tools. Demographic characteristics were parent-reported and weather variables were derived from Environment Canada data. Mixed models were conducted.

**Results:**

In adjusted models (*n* = 241), change in children’s in-care ST (B = -0.07, 95%CI: − 1.43,1.29), LPA (B = 0.08, 95%CI: − 0.89,1.05), and log–transformed MVPA (B = 0.01, 95%CI: − 0.09,0.11) were not significantly different between accreditation and control groups. Age group and the childcare environment were not moderators. Significant increases in BMI Z-score (B = 0.19, 95%CI: 0.03,0.35) and high working memory (OR = 3.24, 95%CI: 1.32,7.97) were observed in the accreditation group and significant increases in expressive vocabulary (B = 3.18, 95%CI: 0.05,6.30) were observed in the control group.

**Conclusions:**

The new accreditation criterion may not significantly change physical activity or ST in childcare settings and therefore may not explain findings for BMI Z-scores and cognitive development. Additional training and resources may be needed.

**Supplementary Information:**

The online version contains supplementary material available at 10.1186/s12889-022-12888-5.

## Background

Physical activity can contribute to healthy physical, cognitive, social–emotional growth and development in early childhood and beyond [1]. Whereas high levels of sedentary behaviour, in particular screen time, can hinder healthy growth and development [2]. Several studies [3–8] have reported that few toddlers (aged 1 to 2 years) and preschoolers (age 3 to 5 years) meet national and international 24-h Movement Guidelines that include recommendations for physical activity, sedentary behaviour, and sleep [9–12]. Therefore, understanding how to best intervene to improve these movement behaviours is needed to support a healthy start for young children.

Targeting physical activity and sedentary behaviour in childcare settings has the potential to improve health behaviours in many children [13, 14]. In Canada, approximately 60%, equating to approximately 1.4 million, children aged 0 to 5 years attend non-parental care [13]. Therefore, having state or provincial policies pertaining to physical activity and sedentary behaviour in childcare settings may be one strategy to reach a large proportion of children [14]. Though these state and provincial policies exist in various jurisdictions, internationally little is known regarding the effectiveness of these policies in changing children’s behaviours [14]. As a result, understanding the effectiveness of state, or provincial childcare policy has been identified as a top 10 research priority for promoting physical activity in preschoolers [14]. In Canada, each province is responsible for their own regulations of childcare settings but most provinces and territories do not have a sedentary behaviour policy or a policy specific to physical activity [15, 16].

In December 2013, the province of Alberta, Canada introduced new accreditation standards for childcare settings (i.e., day care programs, out-of-school care programs and family day home agencies) to be phased in until 2019 that included a new physical activity and sedentary behaviour standard criterion and related indicators [17]. Alberta is the only province or territory in Canada to implement an accreditation program with standards of excellence that exceed provincial regulations [17]. These government-driven criteria provided a unique opportunity to study a naturally occurring provincial policy change regarding physical activity and sedentary behaviour. In 2013/2014, pilot data were collected in 86 children aged 19–60 months from 8 childcare centres in Alberta at baseline and 6 months after the implementation of the new standards. Moderate- to vigorous-intensity physical activity (MVPA; 1.7 min/hr) significantly increased and sedentary time (ST; 2.1 min/hr) and age- and sex-specific body mass index (BMI) z-scores (0.18 SD) significantly decreased in toddlers but not in preschoolers [18]. However, the pilot study did not have a control group and process measures were not collected; therefore, findings should be interpreted with caution. Additionally, the pilot study only examined the impact of the policy on one health indicator (i.e, BMI z-scores). Finally, the role of the childcare environment and childcare educators in this policy change were not explored.

The primary objective of this study was to examine if changes in children’s in-care physical activity and ST differed between centres in and around Edmonton, Alberta, Canada implementing the new standards (accreditation group) compared to non-accredited control centres in and around Ottawa, Ontario, Canada (control group). The secondary objectives were to examine: 1) whether baseline age group (toddler [19–35 months], preschooler [36–60 months]) or the childcare environment moderated any group (accreditation vs. control) differences in change for in-care physical activity or ST, 2) if changes in children’s BMI z-scores or cognitive development differed between accreditation and control groups, and 3) if changes in the perceived strategies used and barriers faced by educators to promote physical activity and minimize sedentary behaviour differed between accreditation and control groups.

## Methods

### Accreditation standards

The Alberta childcare accreditation program, which was developed by the Government of Alberta in consultation with relevant childcare stakeholders, is voluntary but has historically had a high participation rate [17]. The accreditation process takes several months to complete and there is variability between centres on the timeframe for completion. The process starts with applying for pre-accreditation followed by a self-evaluation, the development of a quality enhancement plan, and the implementation of strategies within the plan to meet the standards. Once the strategies have been implemented and supporting documentation has been compiled, a formal on-site evaluation is requested [17]. Childcare programs can access support from coaches when developing their quality enhancement plan.

The accreditation program has existed since 2004, but in 2013 the Alberta government introduced new accreditation standards for childcare settings to be phased in until 2019. The previous accreditation standards and provincial childcare legislation did not mention physical activity or sedentary behaviour [15]. However, a new physical activity and sedentary behaviour standard criterion and related indicators were included in the new accreditation standards. Specifically, Alberta Childcare Accreditation Program Quality Standards included six standards, each with several criteria and related indicators. Standard 2 in the new document (“Program planning and practices support every child’s optimal development in an inclusive early learning and care environment that incorporates the value and importance of play”) includes criterion 2.2, “Child care programs promote physical wellness in all children and incorporate physical literacy in everyday programming” [17]. This criterion includes seven indicators, and the indicator this study primarily focused on was, “promote physical activity and minimize the time that children are sedentary” [17].

### Participants and procedures

The supporting Healthy AcTive CHildcare settings study used a quasi-experimental two-group pre-post design. Participants were children aged 19 to 60 months, and their educators from Canadian childcare centres in and around Edmonton, Alberta (accreditation group) and Ottawa, Ontario (control group). Due to logistical reasons (i.e., the locations of the research team and the number of trips needed to participating centres) data were collected in only one city and its surrounding area in both provinces. Ottawa was selected as the control group because its population size is very similar to Edmonton (Edmonton: 932,546; Ottawa: 934,243 [19]) and no provincial policy changes that would impact physical activity and sedentary behaviour in childcare settings were expected for the course of the study. Children under 19 months of age who typically attend infant programs in Canadian childcare centres were excluded because some children in this age group are not walking independently making it challenging to accurately measure our primary outcome described below [20]. Data collection occurred in two phases, with half of accreditation and control centres participating in 2017/2018 and the other half participating in 2018/2019. Logistically, this ensured at both phases, baseline data were collected between October and early December, and follow-up data were collected approximately 6 months later between April and June. Similar to our pilot study, these time points were selected to minimize seasonal effects of winter and summer and minimize loss to follow-up due to summer vacations and transitions to kindergarten [21, 22].

For childcare centres to be eligible for the study, they had to be licensed with full-time toddler and preschool programs. Childcare centres in the accreditation group were also required to be starting the process of meeting requirements for the updated accreditation program (i.e., had recently applied for pre-accreditation in the applicable phase of data collection) and not be affiliated with another childcare centre that had already completed the program. When contact was made with the director, a total of 12/18 childcare centres in the accreditation group and 8/42 in the control group agreed to participate. Data could not be included from one childcare centre in Edmonton because they dropped out mid- study for unknown reasons, leaving 11 childcare centres in the accreditation group. The lower participation rate in the control group may be due to the lower interest in the study topic of Alberta policy. Both accreditation and control group directors were aware we were focusing on an accreditation criterion related to active behaviours. After completing the study, all childcare centres received a detailed report and $100 resource/equipment of their choice.

To be eligible for participation in the study, children had to be enrolled full-time, aged 19 to 60 months, and not expecting to change childcare arrangements for the duration of the study. A total of 269 children (accreditation: 141; control: 128) had a parent agree on their behalf to participate (participation rate: total = 47%; accreditatio*n* = 67%; control = 36%). However, after excluding 16 ineligible children (age: *n* = 7, part-time: *n* = 7, leaving centre *n* = 2), there were 253 eligible children for the study. To be eligible for participation in the study, educators had to work with children in the eligible age range and not anticipate leaving their job for the duration of the study. A total of 83 educators (accreditation: 44; control: 39) agreed to participate in the study (participation rate: total = 69%; accreditation = 88%; control =55%). However, after excluding 3 ineligible educators (educator in infant age group: *n* = 1, leaving the centre *n* = 2), there were 80 eligible educators. Ethics approvals were obtained from the University of Alberta and the University of Ottawa Research Ethics Boards. Written informed consent was obtained from all directors and participating educators as well as from parents/guardians for all participating children.

Data collection at baseline and 6 month follow-up in the accreditation and control groups included questionnaires for directors, educators as well as a one-day environmental assessment by research staff. The director and educator questionnaires both included questions on the strategies and barriers to physical activity and sedentary behaviour in childcare settings, awareness and familiarity of the Alberta Child Care Accreditation Standards, and demographic characteristics. The director questionnaire also included questions on the characteristics of their centre and experiences of the children in their care. The parent questionnaire included questions on demographic characteristics and their children’s activities outside of childcare. Additionally, at both time points, children wore accelerometers and children’s height and weight, and cognitive development were measured. However, cognitive development assessments were not conducted in toddler rooms, since the assessments are only validated for preschool-aged children [23].

### Process evaluation measures

Three process evaluation measures were captured, including awareness and familiarity of the Alberta Child Care Accreditation Standards (released in December 2013) as well as stage of accreditation. Awareness and familiarity were assessed with one question each at baseline and follow-up in the educator and director questionnaires for both groups. The awareness question was answered with a dichotomous scale that had yes and no response options, and the familiarity question was answered with a Likert scale that had 5 response options ranging from extremely familiar to not at all familiar. Stage of accreditation was assessed at follow-up in the accreditation group by asking directors.

### Primary outcome measures

#### In-care ST and physical activity

Children’s in-care ST and physical activity were measured with waist-worn wGT3X-BT ActiGraph accelerometers for six consecutive weekdays. Childcare staff were provided with verbal and written instructions regarding accelerometer procedures, including putting assigned accelerometer belts on children’s right hip when they arrived in the morning and taking them off before the children went home. Data were downloaded in 15-s epochs. Non-wear time data, defined as ≥80 consecutive 15-s intervals of zero counts (equivalent to ≥20 min of consecutive zero counts, were removed [24]). For children who napped, it was assumed that nap time data were removed with the non-wear time definition. To be included in the analyses, children were required to have ≥3 days with ≥240 total 15-s intervals (equivalent to ≥1 h) of wear time. This definition maximized the sample of children and has been used before in previous childcare studies [25, 26]. Wear time data were classified into ST (≤25 counts/15-s), light-intensity physical activity (LPA; 26–419 counts/15-s), and moderate- to vigorous-intensity physical activity (MVPA; ≥420 counts/15-s) based on validated cut-points for this age group [27–29]. Average minutes per hour of ST, LPA, and MVPA were calculated. SAS version 9.4 (SAS Institute, Cary, NC, USA) was used to conduct all accelerometer data reduction.

### Secondary outcome measures

#### Body Mass Index (BMI)

At both baseline and follow-up, trained research staff measured children’s height to the nearest 0.1 cm using a portable stadiometer and their weight to the nearest 0.1 kg using a calibrated scale. Shoes and bulky clothing were removed prior to the measurements. Height and weight were measured twice unless there was a > 0.2 cm/kg difference between the two measurements then a third measurement was taken. An average of the two closest measurements were recorded. Age- and sex-specific BMI z-scores were calculated based on World Health Organization growth standards [30].

#### Cognitive development

Cognitive development was assessed in preschool rooms only by trained research staff at both baseline and follow-up using two iPad-based tasks from the Early Years Toolbox [23]. The Mr. Ant task measured the working memory aspect of executive functioning, and the Expressive Vocabulary task measured the expressive language aspect of language development [23]. Mr. Ant raw scores had a possible range of 1–8 and Expressive Vocabulary raw scores has a possible range of 0–45. A previous study has reported on psychometric properties for these tasks in 3–5 year olds [23]. Specifically, for convergent validity, significant correlations with medium to large effect sizes [31] were observed for comparable tasks in the National Institute of Health (NIH) Toolbox and British Ability Scales (*r* = 0.46–0.60) [23]. Similar to this previous study [23], good internal consistency reliability [32] was observed in our sample for Expressive Vocabulary (Baseline Cronbach’s α = 0.92). Since the Early Years Toolbox was developed for preschoolers aged 3 to 5 years, cognitive development data were excluded if children were less than 36 months of age in the preschool room.

#### Educator perceptions of strategies and barriers

Strategies and barriers for physical activity and sedentary behaviour in childcare settings were measured with 6 questions at both baseline and follow-up in the HATCH educator questionnaire. The questions were developed by the research team and advisory committee, which included key childcare stakeholders, based primarily on observations and director feedback from the pilot study that informed the present study [18]. Strategies to support regular physical activity were measured with 13 items and barriers were measured with 11 items. Strategies to limit extended periods of sedentary behaviour were measured with 8 items and barriers were measured with 6 items. Strategies to limit screen time were measured with 7 items and barriers were measured with 6 items. All questions also had space for other strategies or barriers. All items were answered with a Likert scale that had 5 response options ranging from never to always. An average score was calculated for each subscale based on the mean of available item scores. Except for strategies to limit extended periods of sedentary behaviour (α = 0.60), good internal consistency reliability [32] was observed for all questions at baseline (α = 0.73–0.90) in educators that had complete data on all items of the subscale (*n* = 50–64). The items were derived from the research team and took into account feedback from key stakeholders in the Alberta childcare sector.

#### Potential moderators

Children’s baseline age group and the childcare environment were considered as potential moderators. Children’s baseline age was calculated based on the first date of accelerometer data collection at baseline and their birth date as reported in the baseline HATCH parent questionnaire. Children’s age in months was categorized into toddler (19–35 months) and preschooler (36–60 months) age groups. The childcare environment was objectively assessed at baseline and follow-up during a one-day observation by a trained researcher using three tools: 1) Environment and Policy Assessment and Observation (EPAO [33]; 2017 version; physical activity component only), 2) Movement Environment Rating Scale (MOVERS) [34, 35], and the Children’s Physical Environments Rating Scale (revised version 5; CPERS5; part C and D only) [36, 37]. During pre-study training, this researcher along with another trained researcher achieved inter-rater agreement above 85% for all tools (85.2% – EPAO and MOVERS, 96.2% – CPERS5) after assessing two childcare centres. The one-day observations were scheduled on a typical day for each centre and focused on preschool rooms and areas because one of the tools (EPAO) has only been validated in preschoolers [33, 38]. Due to low variability of CPERS5 scores observed between centres and low internal consistency for part C, this tool was not included in the present study.

The physical activity component of the EPAO has 13 subscales, which include several aggregated items related to physical activity, screen time, and/or outdoor play and learning. The subscale scores were added to create an overall EPAO physical activity score, which could range from 0 to 39 points. A higher score indicates higher compliance with best practices. To our knowledge, psychometric properties have not been previously reported for the slightly modified EPAO 2017 version. However, previous research on the original EPAO has found similar inter–rater agreement as the present study (87.3% – observation, 79.3% – document review) [33]. MOVERS includes 11 items, which aggregate into 4 subscales related to physical development, movement, and practice. Each item was rated on a scale from 1 (inadequate) to 7 (excellent), and an average of item scores were calculated to create a MOVERS total score [34]. A previous study has reported on the reliability (test-retest: intraclass correlation coefficient (ICC) = 0.96, Weighted Kappa = 0.90, percentage agreement = 69–100%; internal consistency: Cronbach’s alpha = 0.89–0.94) and validity (concurrent validity with some sub-areas of the EPAO and Sustained Shared Thinking and Emotional Wellbeing (SSTEW) scale: Spearman’s rho = 0.57–0.87) of this tool [35].

#### Potential covariates

Children’s age (months), sex, and race/ethnicity as well as parental education were considered as potential covariates in statistical models for the primary outcomes and child secondary outcomes (BMI z-score, cognitive development). Additionally, weather variables including, mean temperature, and mean precipitation were considered as potential covariates in statistical models for the primary outcomes. Children’s birth date, sex, race/ethnicity, and parental education were reported in the baseline HATCH parent questionnaire. Questions were adopted from the Canadian Health Measures Survey [39]. Children’s age at baseline and follow-up were calculated as described above in the potential moderators section. Children’s race/ethnicity was categorized as Caucasian or other (Aboriginal, South Asian, Chinese, African, Filipino, Latin American, Arab, Southeast Asian, West Asian, Korean, Japanese, and other race/ethnicity) based on the frequency distribution. Parental education was categorized as below bachelor level (less than high school diploma, high school diploma, college or trade certificate or diploma), bachelor’s degree, or above bachelor level based on the frequency distribution. Mean temperature and mean precipitation were calculated at baseline and follow-up by averaging the mean daily temperature (i.e., average of the maximum and minimum temperature during a day) and daily total precipitation at the main Edmonton or Ottawa weather stations for the 6 days that children wore the accelerometer (Environment Canada).

Educators’ age (months), sex, and race/ethnicity were considered as potential covariates in statistical models for educator secondary outcomes (i.e., educator strategies and barriers). Educators reported on their birth date, sex, and race/ethnicity in the baseline HATCH educator questionnaire. Educator’s age at baseline and follow-up were also calculated using the first date of data collection and the educator’s birth date. Race/ethnicity was also categorized as Caucasian or other.

### Statistical analysis

Statistical analyses were performed using SPSS version 26.0 (SPSS Inc., Chicago, IL, USA). Descriptive statistics were calculated for child, educator, weather, and childcare centre environment variables at baseline and follow-up (where applicable). T-tests or non-parametric equivalent (Mann-Whitney U Tests) and chi-squared tests were conducted to determine if baseline group differences (accreditation vs. control), and if applicable follow-up group differences, existed for the above-mentioned variables. Additionally, these tests were used to compare child age (months), sex, and group (accreditation and control) variables between children who had baseline and follow-up data and children who had only baseline or follow-up data for in-care ST and physical activity. Finally, descriptive statistics were calculated, and Mann-Whitney U Tests and chi-squared tests were conducted to compare baseline and follow-up process evaluation measures between groups (accreditation vs. control) for both directors and educators.

Statistical assumptions for linear mixed models were assessed by visual inspection. MVPA was log- transformed to meet the assumption of normality. Data for working memory was also not normally distributed but transformations did not improve the distribution. Therefore, working memory was categorized as a dichotomous variable based on the median score (< 1 [range: 0.00–0.67] and ≥ 1 [range:1–3]) [40]. Additionally, LPA and log MVPA were truncated for one participant because they were identified as outliers according to pre-specified criteria [41]. Linear mixed models were conducted to estimate marginal means and standard errors at baseline and follow-up as well as mean differences and standard errors for each outcome variable within each group (accreditation and control). In model 1, the clustering effect of childcare centres and the repeated effect of time were included as random intercepts, and group (accreditation vs. control), time (follow-up vs. baseline), and time*group were included as fixed effects. Model 2 also included covariates as fixed effects where baseline differences and applicable follow-up differences were observed between accreditation and control groups.

To address the primary study objective, linear mixed models were conducted separately for ST, LPA, and log MVPA. In model 1, the clustering effect of childcare centres and the repeated effect of time were included as random intercepts, and group, time, time*group were included as fixed effects. Additionally, in model 2, covariates were included as fixed effects where group (accreditation vs. control) baseline and applicable follow-up differences were observed. If the interaction term (time*group) was significant, stratified analyses were conducted to examine the change in the primary outcome (follow-up vs. baseline) separately in the accreditation and control groups. If the interaction term was not significant, then models were rerun with the interaction term removed to examine the change in the primary outcome (follow-up vs. baseline) in the entire sample, with the accreditation and control groups combined. For these models, additional covariates were considered. Specifically, if a significant difference was not observed between accreditation and control groups but was observed between baseline and follow-up time points, the covariate was included in the model.

To address the first secondary objective, child baseline age group or the childcare environment and a three-way interaction term between group (accreditation vs. control), time (follow-up vs. baseline), and baseline age group (preschoolers vs. toddlers) or continuous environment data were added to the model 1 and model 2 as fixed effects. Once again, if an interaction term was significant, stratified analyses were conducted. To address the second and third secondary objectives, the same modelling strategy was used that addressed the primary objective, except generalized linear mixed models were used for working memory, and a different set of potential covariates were considered. For all mixed models, participants were included if they had data on the model outcome at baseline and/or follow-up, and for model 2 if they also had complete covariate data for that model. Sensitivity analyses were conducted by rerunning the models in the sample of children that had complete data at both points. Additionally, models for the primary outcome were rerun based on the accreditation status at follow-up. Statistical significance was set a priori at *p* < 0.05.

## Results

**Fig. 1:**
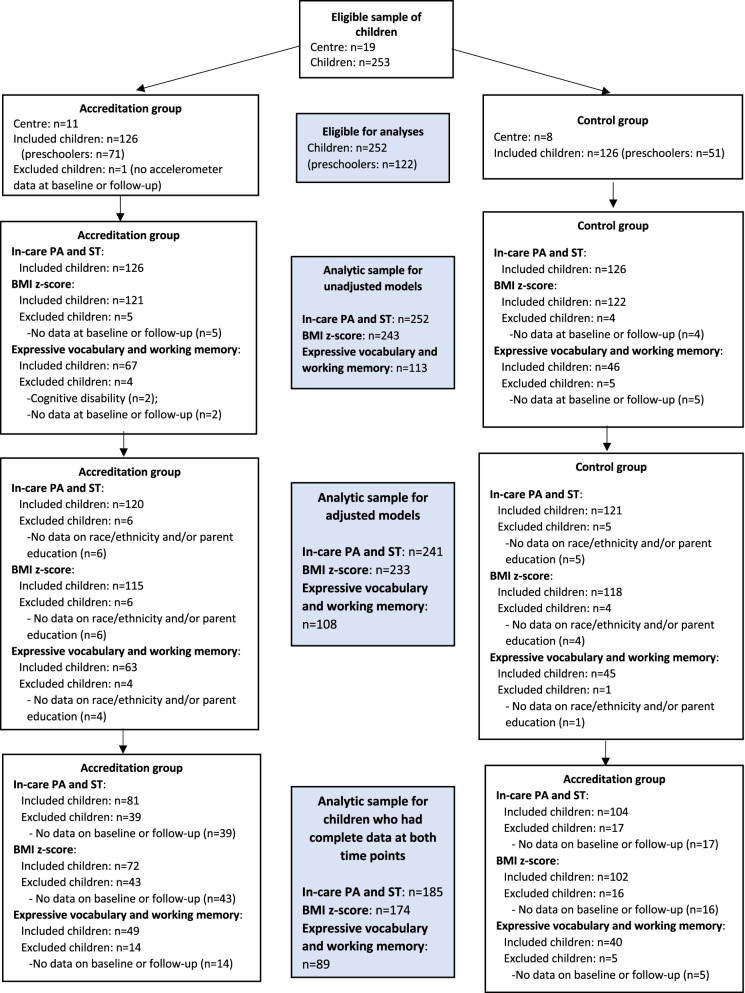
Flowchart of eligible children. BMI, body mass index; PA, physical activity; ST, sedentary time

**Fig. 2:**
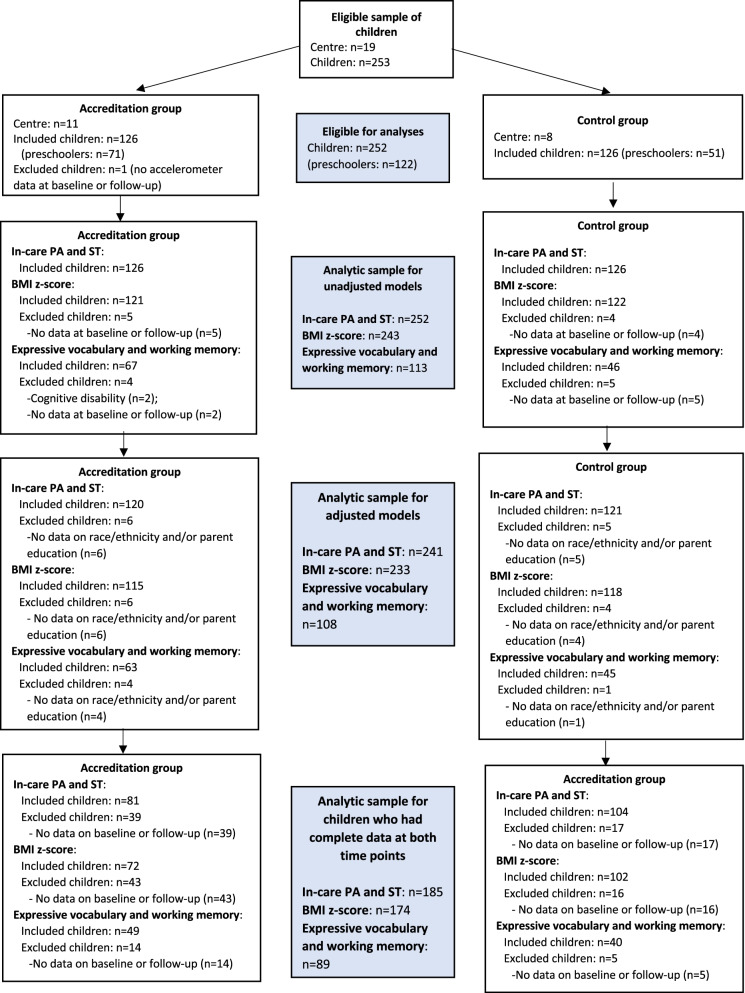
Flowchart of eligible educators. PA, physical activity; SB, sedentary behaviour

Of the 253 eligible children from 19 centres, 252 children (accreditation: *n* = 126; control: *n* = 126) provided data at baseline and/or follow-up on in-care ST and physical activity. A participant flowchart is provided in Fig. 1. Of the 80 eligible educators, 72 (accreditation: *n* = 33; control: *n* = 39) had data at baseline and/or follow-up on strategies and barriers for physical activity and sedentary behaviour in childcare settings (see Fig. 2). There was no significant age or sex differences between children who had data for in-care ST and physical activity at both time points (*n* = 185) compared to children who had data at baseline or follow-up only (*n* = 67). However, the proportion of children from the accreditation group was significantly higher in children with data at a single time point (67%) compared to children with data at both time points (44%). The average number of valid accelerometer days was significantly higher in the control group compared to the accreditation group at baseline (accreditation: 5.1 ± 1.0 days; control: 5.6 ± 0.9 days) and follow-up (accreditation: 5.1 ± 1.0 days; control: 5.6 ± 0.7 days) in the sample of children that had valid data at each time point. Similar findings were observed for average daily wear time at follow-up (accreditation: 6.3 ± 1.8 h/day; control: 7.0 ± 1.1 h/day). However, no significant differences were observed for average daily wear time at baseline (accreditation: 6.4 ± 1.5 h/day; control: 6.6 ± 0.4 h/day).

Participant characteristics for children and educators are shown in Table 1. Children in the accreditation group were significantly older at baseline and follow-up compared to the control group, and significant differences between accreditation and control groups were observed for child race/ethnicity and parental education at baseline. Consequently, in fully-adjusted models (model 2) for the primary outcomes and child secondary outcomes, baseline and follow-up child age was included as a time-varying covariate, and baseline child race/ethnicity and parental education were included as time-constant covariates. Additionally, mean precipitation at baseline and follow-up was significantly higher in the control group compared to the accreditation group and was included in fully adjusted models (model 2) for the primary outcomes as a time-varying covariate. There were no significant differences in educators’ characteristics between the accreditation and control groups. As such, only model 1 analyses were performed for educator secondary outcomes.

**Table 1:** Descriptive information and group (accreditation vs. control) differences

	Baseline			Follow-up		
	Accreditation Group	Control Group	*P*-value	Accreditation Group	Control Group	*P*-value
**Children’s characteristics**	**(*n*** **= 126)**	**(*n*** **= 126)**	─	**(*n*** **= 126)**	**(*n*** **= 126)**	─
Age (years; M ± SD)^a^	(*n* = 126)	(*n* = 126)	─	(*n* = 126)	(*n* = 126)	─
	3.1 ± 0.9	2.8 ± 0.6	**0.003**	3.7 ± 0.9	3.3 ± 0.7	**< 0.002**
Sex (%)^b^	(*n* = 126)	(*n* = 126)	─	(*n* = 126)	(*n* = 126)	─
Male	55.6	46.8	0.166	─	─	─
Female	44.4	53.2		─	─	─
Race/ethnicity (%)^b^	(*n* = 122)	(*n* = 124)	─	─	─	─
Caucasian	43.4	61.3	**0.005**	─	─	─
Other	56.6	38.7		─	─	─
Missing data	(*n* = 4)	(*n* = 2)	─	─	─	─
Parental education (%)^b^	(*n* = 121)	(*n* = 121)	─	─	─	─
Below bachelor level	52.1	20.7	**< 0.001**	─	─	─
Bachelor’s degree	24.8	34.7		─	─	─
Above bachelor level	23.1	44.6		─	─	─
Missing data	(*n* = 5)	(*n* = 5)	─	─	─	─
**Weather**	**(*n*** **= 11)**	**(*n*** **= 8)**	─	**(*n*** **= 11)**	**(*n*** **= 8)**	─
Mean temperature (°C, M ± SD)^a,c^	−2.8 ± 5.6	−2.7 ± 5.3	0.983	9.2 ± 5.9	13.3 ± 3.7	0.103
Mean precipitation (mm, M ± SD)^a^	0.5 ± 0.8	2.3 ± 1.7	**0.005**	1.0 ± 1.3	3.2 ± 2.4	**0.026**
**Childcare characteristics**	**(*n*** **= 11)**	**(*n*** **= 8)**	─	**(*n*** **= 11)**	**(*n*** **= 8)**	─
MOVERS score (1–7; M ± SD)^a^	3.1 ± 1.0	3.8 ± 1.1	0.216	3.9 ± 0.8	4.2 ± 0.9	0.514
EPAO PA score (0–39; M ± SD)^a^	20.9 ± 2.2	21.8 ± 1.3	0.354	23.1 ± 3.10	23.6 ± 1.1	0.686
**Educators’ characteristics**	**(*n*** **= 33)**	**(*n*** **= 39)**	─	**(*n*** **= 33)**	**(*n*** **= 39)**	─
Age (Years; M ± SD)^a^	(*n* = 32)	(*n* = 36)	─	(*n* = 32)	(*n* = 36)	─
	37.9 ± 11.4	37.1 ± 10.7	0.806	38.3 ± 11.4	37.7 ± 11.1	0.849
Missing data	(*n* = 1)	(*n* = 3)	─	(*n* = 1)	(*n* = 3)	─
Sex (%)^d^	(*n* = 33)	(*n* = 39)	─	(*n* = 33)	(*n* = 39)	─
Male	7.7	3.0	─	─	─	─
Female	92.3	97.0		─	─	─
Race/ethnicity (*n* = 71)^b^	(*n* = 33)	(*n* = 38)	─	─	─	─
Caucasian	72.7	50	0.051	─	─	─
Other	27.3	50		─	─	─
Missing data	─	(*n* = 1)	─	─	─	─

*Abbreviations*: *M* Mean, *SD* Standard deviation, *MOVERS* Movement Environment Rating Scale, *EPAO* Environment and Policy Assessment and Observation, *PA* Physical activity

Bold fonts indicate *p* < 0.05

^a^Mann-Whitney U Tests (child age, mean precipitation, educator age) and t-tests (mean temperature, MOVERS total score, EPAO PA score) were used to compare continuous data between accreditation and control groups at baseline and follow-up

^b^Chi-square tests were used to compare categorical data (child sex, child race/ethnicity, parental education, and educator race/ethnicity) between accreditation and control groups at baseline

^c^Mean temperature was significantly different between baseline and follow-up in the accreditation and control groups

^d^It was not possible to test for sex differences between groups because less than 5 educators were male in both groups (accreditation group: *n* = 1; control group: *n* = 3)

Findings for awareness and familiarity of the Alberta Child Care Accreditation Standards are included in Table 2. Compared to the control group, directors and educators in the accreditation group had significantly higher awareness and familiarity at baseline and follow-up, except for educator awareness at follow-up, which was non-significant (Accreditation: 59%; Control; 39%; *p* = 0.052). In terms of the childcare accreditation status that was assessed at follow-up there was variation as expected. For the 11 centres in the accreditation group, one centre was now accredited, one centre had their accreditation site visit and received notice 1 month later that they passed the evaluation and were officially accredited, and three centres were officially accredited 3 to 4 months after follow-up data collection suggesting their site visit was scheduled shortly after the data collection session. In terms of the remaining six centres, two centres were accredited 5 months after follow-up data collection, two centres were accredited 9 to 10 months after follow-up data collection, and two centres did not become accredited.

**Table 2:** Awareness and familiarity of the Alberta Child Care Accreditation Standards (released in December 2013) in directors and educators for accreditation and control groups

	Time	Group	n	Awareness (%)	*P* value^*^	n	Familiarity (M ± SD)^c^	*P* value^*^^*^
Directors (*n* = 19)^a^	Baseline	Accreditation	10	100.0	**< 0.001**	10	4.0 ± 0.8	**< 0.001**
		Control	8	0		8	1.0 ± 0.0	
	Follow-up	Accreditation	11	100.0	**< 0.001**	11	4.1 ± 0.7	**< 0.001**
		Control	8	0		8	1.0 ± 0.0	
Educators (*n* = 72)^b^	Baseline	Accreditation	32	50.0	**0.002**	27	2.9 ± 1.6	**< 0.001**
		Control	39	15.4		39	1.2 ± 0.6	
	Follow-up	Accreditation	22	59.1	0.052	18	3.1 ± 1.5	**0.001**
		Control	31	32.3		31	1.7 ± 1.1	

*Abbreviations*: *M* Mean, *SD* Standard deviation

Bold fonts indicate *p* < 0.05

^a^Sample size for directors included in the analytic sample: accreditation group (*n* = 11), control group (*n* = 8). Missing data at baseline and follow-up existed for one director in the accreditation group

^b^Sample size for educators included in the analytic sample: accreditation group (*n* = 33), control group (*n* = 39). Missing data at baseline and follow-up existed for educators in the accreditation group and at follow-up for the control group

^c^Higher M ± SD scores (Range:1–5) indicate more familiarity with the Alberta Child Care Accreditation Standards (released in December 2013)

^*^Chi-square tests were used to examine the differences in awareness between accreditation and control groups

^*^^*^ Mann-Whitney U Tests were used to examine the differences in familiarity between accreditation and control groups

Findings for the primary objective are presented in Table 3. No significant differences for change in children’s in-care sedentary time (B = -0.06; 95%CI: − 1.39,1.27), LPA (B = -0.03; 95%CI: − 0.99,0.93) and log MVPA (B = 0.01; 95%CI: − 0.09,0.11) were observed between groups in model 1. Similar findings were observed for model 2. In the sensitivity analysis of the sample of children who had data at both time points, beta coefficients were larger but no significant differences in change were observed (data not shown). Additionally, no significant differences were observed when only accreditation centres that became accredited (*n* = 9) were included or when only accreditation centres that became accredited within 3–4 months of follow-up data collection were included (*n* = 5; data not shown). When the interaction term was removed, a significant change was observed for sedentary time (B = − 1.38; 95%CI:-2.04,-0.72), LPA (B = 0.56: 95%CI:0.09,1.04), and log MVPA (B = 0.12: 95%CI: 0.07,0.17) in the entire sample in model 1. However, these associations were attenuated and became non-significant in model 2, when mean temperature was added as a covariate (supplementary Table 1). Mean temperature was added to model 2 because it was significantly higher at baseline compared to follow-up in the accreditation and control groups.

**Table 3:** Differences for change in children’s in-care sedentary time and physical activity (follow-up vs. baseline) between groups (accreditation vs. control)

Outcome variables	Groups	Baseline	Follow-up	Follow-up vs. baseline	Group*time	
**Model 1**						
		Estimated marginal means (SE)		Mean difference (SE)	B (95%CI)^a^	*P* value
Sedentary time (min/h)	Accreditation (*n* = 126)	31.87 (0.43)	30.45 (0.47)	−1.41 (0.50)	− 0.06(− 1.39,1.27)	0.802
	Control (*n* = 126)	31.96 (0.43)	30.61 (0.42)	−1.35 (0.46)		
LPA (min/h)	Accreditation (*n* = 126)	21.23 (0.30)	21.78 (0.33)	0.55 (0.36)	−0.03(− 0.99,0.93)	0.953
	Control (*n* = 126)	22.00 (0.30)	22.57 (0.30)	0.58 (0.33)		
MVPA (min/h)	Accreditation (*n* = 126)	6.90 (0.21)	7.78 (0.28)	0.88 (0.27)	0.01(−0.09,0.11)^b^	0.802
	Control (*n* = 126)	6.04 (0.21)	6.81 (0.25)	0.77 (0.24)		
**Model 2**						
		Estimated marginal means (SE)		Mean difference (SE)	B (95%CI)^a^	*P* value
Sedentary time (min/h)	Accreditation (*n* = 120)	31.97 (0.48)	30.72 (0.52)	−1.25 (0.55)	−0.07(−1.43,1.29)	0.918
	Control (*n* = 121)	31.71 (0.47)	30.53 (0.48)	−1.18 (0.50)		
LPA (min/h)	Accreditation (*n* = 120)	21.41 (0.34)	22.12 (0.37)	0.71 (0.39)	0.08(−0.89,1.05)	0.873
	Control (*n* = 121)	21.75 (0.33)	22.39 (0.34)	0.64 (0.36)		
MVPA (min/h)	Accreditation (*n* = 120)	6.65 (0.23)	7.23 (0.29)	0.58 (0.29)	0.01(−0.09,0.11)^b^	0.891
	Control (*n* = 121)	6.49 (0.23)	7.03 (0.27)	0.54 (0.26)		

*Abbreviations*: *B* unstandardized beta coefficient, *SE* Standard error, *CI* Confidence interval, *LPA* Light-intensity physical activity, *MVPA* Moderate-to vigorous-intensity physical activity

In model 1, the clustering effect of childcare centers and the repeated effect of time were included as random intercepts, and time, group, and time*group were included as fixed effects

In model 2, the clustering effect of childcare centers and the repeated effect of time were included as random intercepts, and time, group, time*group, and covariates (child age, child race/ethnicity, parental education, mean precipitation) were included as fixed effects

^a^Estimate for the interaction term between group (accreditation vs. control) and time (follow-up vs. baseline) and can be interpreted as the differences in change between groups for each outcome variable

^b^Given the non-normal distribution of data for MVPA, the unstandardized beta coefficient was calculated for Log (MVPA)

The findings of the secondary objectives are shown in Tables 4, 5 and 6. Baseline age group and childcare environment did not significantly moderate the differences for change in children’s in-care ST, LPA, and log MVPA between groups in models 1 or 2 (Table 4). Similar findings were observed in the sensitivity analysis of the sample children who had data at both time points (data not shown). The change in children’s BMI z-scores, expressive vocabulary, and working memory were significantly different between groups in models 1 and 2 (Table 5) and for the sensitivity analysis (data not shown). For stratified analysis, significant increases in BMI Z-score (B = 0.19, 95%CI: 0.03,0.35) and high working memory (OR = 3.24, 95%CI: 1.32,7.97) were only observed in the accreditation group and significant increases in expressive vocabulary (B = 3.18, 95%CI: 0.05,6.30) were only observed in the control group for model 2 (supplementary Table 2). Finally, no significant group differences were observed for change in educators’ strategies and barriers related to physical activity and sedentary behaviour in childcare settings (Table 6).

**Table 4:** The moderating effects of age group and childcare environment on the differences for change in-care physical activity and sedentary time (follow-up vs. baseline) between groups (accreditation vs. control)

Outcome variables	Moderators					
	Age group		MOVERS total score		EPAO PA score	
**Model 1**						
	B (95%CI)^a^	*P* value	B(95%CI)^b^	*P* value	B (95%CI)^b^	*P* value
Sedentary time (min/h; *n* = 252)	1.49(− 1.25,4.23)	0.286	0.60(− 1.34,2.55)	0.541	0.12(− 0.88,1.11)	0.815
LPA (min/h; *n* = 252)	− 0.74(−2.70,1.22)	0.458	−1.12(−2.50,0.25)	0.110	− 0.03(− 0.75,0.68)	0.924
Log (MVPA) (*n* = 252)	− 0.09(− 0.29,0.11)	0.376	0.02(− 0.12,0.17)	0.750	0.75(− 0.95,2.45)	0.386
**Model 2**						
	B (95%CI)^a^	*P* value	B(95%CI)^b^	*P* value	B (95%CI)^b^	*P* value
Sedentary time (min/h; *n* = 241)	−1.42(−4.23,1.38)	0.318	−0.39(− 2.76,1.98)	0.744	0.07(− 0.97,1.11)	0.897
LPA (min/h; *n* = 241)	0.46(−1.53,2.44)	0.651	0.27(−1.40,1.95)	0.747	−0.11(− 0.84,0.63)	0.776
Log (MVPA) (*n* = 241)	0.11(−0.09,0.32)	0.281	−0.05(− 0.23,0.13)	0.594	− 0.02(− 0.10,0.06)	0.637

*Abbreviations*: *B* Unstandardized beta coefficient, *CI* Confidence interval, *LPA* Light-intensity physical activity, *MVPA* Moderate-to vigorous-intensity physical activity, *MOVERS* Movement Environment Rating Scale, *EPAO* Environment and Policy Assessment and Observation, *PA* Physical activity

In model 1, the clustering effect of childcare centers and the repeated effect of time were included as random intercepts, and time, group, and time*group were included as fixed effects

In model 2, the clustering effect of childcare centers and the repeated effect of time were included as random intercepts, and time, group, time*group, and covariates (child age, child race/ethnicity, parental education, and mean precipitation) were included as fixed effects

^a^Beta coefficients for the interaction term between group (accreditation vs. control), time (follow-up vs. baseline), baseline age group (preschooler vs. toddler). ^b^Beta coefficients for the interaction term between group (accreditation vs. control), time (follow-up vs. baseline), and the MOVERS total score (continuous)

^3^ Beta coefficients for the interaction term between group (accreditation vs. control), time (follow-up vs. baseline), and the EPAO PA score (continuous)

**Table 5:** Differences for change in children’s BMI z-scores, vocabulary, and working memory (follow-up vs. baseline) between groups (accreditation vs. control)

Outcome variables	Groups	Baseline	Follow-up	Follow-up vs. baseline	Group*time	
**Model 1**						
		Estimated marginal means (SE)		Mean difference (SE)	B (95%CI)^b^	*P* value
BMI z-score	Accreditation (*n* = 121)	0.56 (0.09)	0.63 (0.10)	0.07 (0.06)	**0.20 (0.05,0.35)**	**0.011**
	Control (*n* = 122)	0.85 (0.09)	0.73 (0.09)	−0.13 (0.05)		
Expressive vocabulary^a^	Accreditation (*n* = 67)	18.01 (1.00)	22.15 (1.02)	4.15 (0.53)	**−1.69(−3.26,-0.13)**	**0.034**
	Control (*n* = 46)	20.24 (1.20)	26.08 (1.22)	5.84 (0.58)		
		Estimated marginal means (SE)		Mean difference (SE)	OR (95%CI)^c^	*P* value
Working memory^a^	Accreditation (*n* = 67)	0.25 (0.06)	0.70 (0.07)	0.45 (0.08)	**5.20 (1.70,15.86)**	**0.004**
	Control (*n* = 46)	0.62 (0.09)	0.69 (0.09)	0.07 (0.09)		
**Model 2**						
		Estimated marginal means (SE)		Mean difference (SE)	B (95%CI)^b^	*P* value
BMI z-score	Accreditation (*n* = 115)	0.51 (0.10)	0.72 (0.11)	0.21 (0.07)	**0.18 (0.03,0.34)**	**0.021**
	Control (*n* = 118)	0.77 (0.10)	0.79 (0.10)	0.03 (0.07)		
Expressive vocabulary^a^	Accreditation (*n* = 63)	19.49 (0.88)	19.47 (1.03)	−0.02 (0.86)	**−1.64(−3.24,-0.05)**	**0.043**
	Control (*n* = 45)	22.82 (1.14)	24.44 (1.07)	1.62 (0.88)		
		Estimated marginal means (SE)		Mean difference (SE)	OR (95%CI)^c^	*P* value
Working memory^a^	Accreditation (*n* = 63)	0.30 (0.07)	0.62 (0.09)	0.32 (0.10)	**5.71 (1.77,18.43)**	**0.004**
	Control (*n* = 45)	0.71 (0.09)	0.61 (0.09)	−0.09 (0.11)		

*Abbreviations*: *B* Unstandardized beta coefficient, *OR* Odds ratio, *SE* Standard error, *CI* Confidence interval, *BMI* Body mass index

Bold font indicates *p* < 0.05

In model 1, the clustering effect of childcare centers and the repeated effect of time were included as random intercepts, and time, group, and time*group were included as fixed effects

In model 2, the clustering effect of childcare centers and the repeated effect of time were included as random intercepts, and time, group, time*group, and covariates (child age, child race/ethnicity, parental education, and mean precipitation) were included as fixed effects

^a^Expressive vocabulary (ranging from 0 to 45) and working memory (value “0” represents score ≤ 1 [range: 0.00–1.00]; value “1” represents score > 1 [range: 1.33–3.67]) were measured in preschool children only

^b^Estimate for the interaction term between group (accreditation vs. control) and time (follow-up vs. baseline) and can be interpreted as the differences in change between groups for each outcome variable

^c^Estimate for the interaction term between group (accreditation vs. control) and time (follow-up vs. baseline) and can be interpreted as the odds of having higher working memory (1 vs. 0) at follow-up (vs. baseline) in the accreditation group compared to the control group

**Table 6:** Differences for change in educators’ strategies and barriers related to physical activity and sedentary behaviour in childcare settings (follow-up vs. baseline) between groups (accreditation vs. control)

Subscales	Groups^a^	Baseline	Follow-up	Follow-up vs. baseline	Group*time	
		Estimated marginal mean (SE)^b^		Mean difference (SE)	B (95%CI)^c^	*P* value
Strategies to support regular physical activity	Accreditation (*n* = 33)	4.40 (0.07)	4.53 (0.08)	0.13 (0.07)	0.13(−0.05,0.32)	0.151
	Control (*n* = 39)	4.25 (0.07)	4.24 (0.07)	−0.002 (0.06)		
Strategies to limit extended periods of sedentary behaviour	Accreditation (*n* = 33)	4.20 (0.10)	4.01 (0.13)	−0.19 (0.13)	−0.29(− 0.62,0.05)	0.092
	Control (*n* = 39)	4.00 (0.09)	4.09 (0.11)	0.09 (0.11)		
Strategies to limit screen time	Accreditation(*n* = 32)	4.35 (0.17)	4.35 (0.14)	−0.22 (0.21)	−0.27(− 0.82,0.27)	0.323
	Control (*n* = 39)	4.25 (0.12)	4.30 (0.15)	0.05 (0.18)		
Barriers in supporting regular physical activity	Accreditation (*n* = 33)	2.27 (0.13)	2.08 (0.20)	−0.19 (0.22)	−0.37(− 0.95,0.20)	0.202
	Control (*n* = 39)	2.06 (0.12)	2.25 (0.16)	0.18 (0.19)		
Barriers in limiting extended periods of sedentary behaviour	Accreditation (*n* = 33)	2.15 (0.11)	1.92 (0.19)	−0.23 (0.20)	−0.37(− 0.88,0.15)	0.160
	Control (*n* = 39)	1.78 (0.10)	1.92 (0.16)	0.14 (0.17)		
Barriers in limiting screen time	Accreditation (*n* = 31)	1.79 (0.09)	1.86 (0.19)	0.07 (0.20)	−0.03(−0.54,0.48)	0.905
	Control (*n* = 38)	1.30 (0.15)	1.20 (0.08)	0.10 (0.16)		

*Abbreviations*: *SE* Standard error, *CI* Confidence interval

In all models the clustering effect of childcare centers and the repeated effect of time were included as random intercepts, and time, group, time*group were included as fixed effects

^a^Sample size for educators included in the analytic sample: accreditation group (*n* = 33), control group (*n* = 39). Missing data existed for educators in the accreditation group for two subscales and in the control group for one subscale

^b^Higher Mean (SE) scores indicate that the strategies used more frequently (Range: 1–5) or the barrier was faced more frequently (Range: 1–5)

^c^Estimate for the interaction term between group (accreditation vs. control) and time (follow-up vs. baseline) and can be interpreted as the differences in change between groups for each subscale

## Discussion

The primary objective of this study was to examine if implementing the new Alberta Childcare Accreditation Program Quality Standards was associated with changes in children’s in-care physical activity and ST. Overall, changes in these behaviours did not differ between children attending centres in and around Edmonton, Alberta, Canada implementing the new standards and children attending non-accredited control centres in and around Ottawa, Ontario, Canada. In terms of secondary objectives, baseline age group and the childcare environment did not appear to moderate changes in children’s in-care physical activity and ST. Additionally, group differences were not observed for changes in the perceived strategies used and barriers faced by educators to promote regular physical activity and minimize sedentary behaviour. Finally, though some group differences were observed for changes in BMI Z-scores and cognitive development, these findings were likely not related to in-care physical activity and ST.

This study builds on and extends our pilot work [18] by including a larger sample and a control group as well as additional measures. In our pilot study, a significant decrease in ST and a significant increase in MVPA were observed among toddlers 6.5 months after participating centres had started the accreditation process. Conversely, a significant increase in ST and a significant decrease in LPA were observed among preschoolers. However, we acknowledged that observed differences in behaviours may be explained by external factors, such as normal growth and development [18]. Our findings in the present study, which demonstrate that changes in children’s in-care physical activity and ST were not significantly different between groups and that differences in change between groups were not moderated by baseline age, confirm the importance of having a control group when examining policy changes. In the present study, a significant effect of time on children’s in-care physical activity and ST was observed in the overall sample in models without covariates, though the observed changes in behaviours were smaller compared to the pilot study [18]. Additionally, once covariates were added to the models, in particular mean temperature, the effect of time was attenuated and no longer significant. A previous study in Alberta childcare centres found that outdoor play, which typically includes more physical activity than indoors [42–44], was significantly lower in the winter (December to March) compared to non-winter (April to November) months [21]. We purposely tried to collect data in non-winter months to minimize seasonal effects on our primary outcomes. Unlike our pilot study, baseline data collection did occur in early December for two childcare centres in the control group. Overall, these findings suggest temperature is an important factor to consider when examining changes in children’s physical activity and ST in childcare centres, especially in regions where there are larger seasonal differences in temperature.

Beyond season and weather, there are a number of reasons why the new accreditation standards may not have been effective at increasing children’s in-care physical activity and decreasing their ST in the present study. For instance, the Alberta Childcare Accreditation Program Quality Standards are comprehensive, and centres that were working towards accreditation for the first time would not have been solely focusing on the criterion related to physical activity and sedentary behaviour, even though this criterion was new. Additionally, not all centres were accredited at follow-up and our other process evaluation measures suggest there were likely some standards, criteria, and/or indicators that directors and educators were less familiar with. Specifically, on average, at follow-up in the accreditation group, directors were “moderately familiar” and educators were “somewhat familiar” with the new accreditation standards as a whole. However, our sensitivity analyses suggest that the variation in accreditation status may not be a key explanation for our null findings. If we assume that centres were familiar with criterion 2.2, given their participation in our study, another explanation for our null findings may be that centres did not have the necessary knowledge or support to make changes that would be substantial enough to impact these behaviours. Specifically, the support that was provided during the accreditation process in the form of coaches was not extensive and did not necessarily cover all standards, rather it depended on the specific needs of a centre. Our findings that there were no significant group differences in changes of educator perceptions suggests the criterion related to physical activity and sedentary behaviour may not have been a major focus of coaching support for participating centres. Additionally, on average, educators reported frequently using strategies and rarely facing barriers to promote physical activity and limit sedentary behaviour. Even though our educator questionnaire captured some key barriers identified in previous literature, such as small spaces, inappropriate clothing, limited equipment, risk of injury, and poor weather [45]. Finally, given the related indicators for the physical activity and sedentary behaviour criterion were somewhat vague and did not include specific benchmarks, it would have been difficult for centres to know whether improvements were needed for this criterion.

Studies examining the effectiveness of state or provincial childcare policy in changing children’s physical activity and sedentary behaviour are limited [28], though findings of the present study can be compared to a similar study in the United States [46]. Specifically, the effectiveness of a new statewide childcare policy of 60 min per day of physical activity in Massachusetts was compared to a control state of Rhode Island. The new policy was part of a larger group of regulations and participating centres were selected from one city in each state. Based on direct observation in cross-sectional samples of 3–5-year-old children, it was found that children’s in-care physical activity, primarily LPA, increased between pre-and post-time points in both groups. However, similar to the present study, the changes were not significantly different between groups. In contrast to Alberta, the new physical activity policy in Massachusetts had a specific benchmark for physical activity (i.e., 60 min per day) [46]. However, it was not clear whether any training was provided to centres when implementing the new policy [46]. After the introduction of the Alberta Childcare Accreditation Program Quality Standards, another provincial policy change regarding physical activity and sedentary behaviour occurred in the Canadian province of British Columbia [47]. Specifically, a mandatory standalone policy outlining standards for active play and sedentary behaviour was introduced in 2016/2017 [47]. Like Massachusetts, there were specific benchmarks included in the policy for physical activity and sedentary behaviour. For instance, a total of 120 min per day of active play and physical movement should be incorporated, including a minimum of 60 min per day of outdoor active play. In terms of sedentary behaviour, screen time should be limited to 30 min or less per day, and children less than 2 years or child in a program for 3 h or less should not be offered screen time [47]. Unique to the British Columbia policy change, an extensive capacity building initiative called Appetite to Play was also implemented to provide childcare providers with the necessary training to make changes to centre-level practice, as well as environmental and policy changes related to the new provincial policy [48]. Evidence to date indicates this initiative increased childcare providers’ knowledge, confidence, and intentions to promote physical activity and physical literacy [48]. Additionally, the policy and initiative has also increased practices and policies regarding physical activity and sedentary behaviour in childcare centres in British Columbia (Tugault-Lafleur et al., Under Review). Future research is needed to determine if the new provincial policy and related initiative also result in changes to children’s in-care physical activity and ST. Based on the evidence thus far, future policy changes in Alberta should consider more specific indicators and more extensive training and support for educators and directors, consistent with the British Columbia model. However, future research is needed to inform evidence-based physical activity and sedentary behaviour recommendations that are specific to childcare settings.

Our findings based on accelerometer-measured behaviours suggest that there may be room for improvement in children’s in-care physical activity and ST. For instance, on average, children in both the accreditation and control groups were spending more than half of their time sedentary at both time points. The Canadian 24-Hour Movement Guidelines [11] as well as guidelines from the World Health Organization [12] recommend replacing ST, in particular time spent restrained and sedentary screen time, with additional MVPA for greater health benefits. Of note, the estimates of ST were lower, and the estimates of physical activity were higher in the present study compared to our pilot study [18]. These discrepancies may be explained by the different accelerometer devices used between studies (i.e., ActiGraph vs. Actical). For instance, a previous study that compared estimates of these behaviours between these devices in 4 and 5-year-olds, with slightly different cut-points than our work, observed similar findings [49]. Specifically, it was reported that the ActiGraph device, which was used in the present study, registered higher estimates of physical activity, whereas the Actical device, which was used in the pilot study, registered higher estimates of ST [49]. Overall, considerable variation exists between studies for estimates of accelerometer-measured in-care physical activity and ST, partly due to different devices and different cut-points [50]. As outlined in a previous HATCH paper that included a subsample of baseline participants [51], the estimates of children’s in-care physical activity and ST observed in the present study are similar with previous childcare research that have used ActiGraph accelerometers with the same cut-points [50].

The findings regarding our secondary outcomes, including BMI Z-score, expressive vocabulary, and working memory are difficult to explain, given there were no significant group differences in changes of children’s in-care physical activity and ST. Additionally, the group differences observed for these health indicators were inconsistent. Our findings for BMI Z-score were also inconsistent with findings from our pilot study, where a significant decrease in BMI Z-score was observed in toddlers [18]. It is important to note that these health indicators could also be impacted by behaviours outside of childcare. Therefore, future research examining these health indicators should consider predicators in both home and childcare settings, where relevant.

Baseline age and the childcare environment did not appear to be moderate group differences in change of physical activity and ST. It is possible we were underpowered for these three-way interactions. A baseline HATCH paper on the association between the childcare environment and physical activity in ST, reported some trends [52]. More specifically, childcare policy was important for toddler behaviours, whereas characteristics of the outdoor play and learning environment (e.g., time, professional development, play yards), curriculum, and pedagogy were important for preschooler behaviours [52]. This aligns with the a systematic review that found that the outdoor environment was the most consistent correlate of both physical activity and sedentary behaviour, in childcare settings [53]. Additionally, a recent call to action to increase developmentally appropriate opportunities for MVPA in all childcare settings identified childcare educators, physical environments, outdoor time, and policy as important targets [54]. Therefore, how these factors interact to change physical activity and ST in childcare settings, and if significant differences exist between age groups, warrants further examination.

Strengths of the study include the objective measures of children’s in care physical activity and ST in a relatively large sample of toddlers and preschoolers. The heights and weights that were used to calculate the BMI Z-score were also objectively measured, and the tasks that were used to assess cognitive development have established reliability and validity [23]. Additionally, the inclusion of a control group and the addition of cognitive development, environment, educator, and process evaluation measures, addressed limitations of our pilot study and enabled a more comprehensive examination of the new accreditation standard criterion. One limitation of the study was not all centres in the accreditation group were officially accredited at the 6-month follow-up. However, findings were consistent in our sensitivity analyses when only accredited centres and centres near the end of the accreditation process (i.e., within 3–4 months of our follow-up time point) were included, representing centres that were accredited, had their site visit, or would be scheduling their site visits shortly after a follow-up data collection. Additionally, if a later follow-up time point occurred in the summer or fall, it is anticipated our loss to follow-up would have been significantly higher due to summer vacations and children leaving the centres for kindergarten. Another limitation of our study is the generalizability of findings to childcare centres throughout the province of Alberta. As previously mentioned, due to logistical reasons we only collected data in one main city and surrounding area in the province, and the sample size of included centres is a small proportion of childcare centres in this region. Additionally, we may have been underpowered for our moderation analyses. Finally, while our models controlled for observed group differences, residual confounding may have occurred due to our quasi-experimental design.

## Conclusions

This study focused on a naturally occurring provincial policy change related to physical activity and ST in childcare centres. Findings suggest the new accreditation standard criterion in Alberta, Canada, regardless of the childcare environment, may not be enough on its own to change these behaviours in toddlers or preschoolers. Also, the new accreditation standard combined with coaching support appeared to have little to no impact on educators’ perceptions related to physical activity and sedentary behaviour. Therefore, additional training and resources regarding centre-level practice, environmental, and policy changes related to physical activity and sedentary behaviour may be needed to support directors and educators implementing similar policy changes. The development of more specific evidence-based recommendations for physical activity and sedentary behaviour within childcare centres should also be considered for future research and subsequent policy changes. Finally, future research is needed to better understand what aspects of childcare and/or home settings may explain our observed findings for BMI Z-scores and cognitive development.

## Supplementary Information

**Additional file 1.**

## Data Availability

The data that support the findings of this study are available on request from the corresponding author [VC]. The data are not publicly available due to limitations of ethical approval involving the participant data and anonymity.

## Abbreviations

B: Unstandardized beta coefficient
BMI: Body Mass Index
CI: Confidence Interval
CPERS5: The Children’s Physical Environments Rating Scale revised version 5
EPAO: Environment and Policy Assessment and Observation
HATCH: The supporting Healthy physical AcTive CHildcare setting study
LPA: Light-intensity Physical Activity
MOVERS: Movement Environment Rating Scale
MVPA: Moderate- to Vigorous-intensity Physical Activity
OR: Odds Ratio
SD: Standard Deviation
SE: Standard Error
ST: Sedentary Time
