# Supplementary material for: The impact of new government childcare accreditation standards on children’s in-care physical activity and sedentary time
Source: BMC Public Health. 2022 Mar 29;22:616. doi: 10.1186/s12889-022-12888-5 (PMC8966317; doi:10.1186/s12889-022-12888-5)
Supplement: Supplementary file 1 — Additional file 1. [file 12889_2022_12888_MOESM1_ESM.docx]

**Supplementary Table 1.** Change in children’s in-care physical activity and sedentary time (follow-up vs. baseline)

| Outcome variables | Models | Baseline | Follow-up | Time | |
| --- | --- | --- | --- | --- | --- |
|  |  | Estimated marginal means (SE) | | B(95%CI) | P value |
| Sedentary time (min/h) | Model 1 (n=252)^1^ | 31.91(0.30) | 30.54(0.32) | **-1.38(-2.04,-0.72)** | **<0.001** |
|  | Model 2 (n=241)^2^ | 31.43(0.37) | 31.11(0.40) | -0.32(-1.44,0.80) | 0.574 |
| LPA(min/h) | Model 1 (n=252)^1^ | 21.62(0.21) | 22.18(0.22) | **0.56(0.09,1.04)** | **0.021** |
|  | Model 2 (n=241)^2^ | 21.84(0.26) | 21.95(0.29) | 0.11(-0.68,0.91) | 0.781 |
| MVPA(min/h) | Model 1 (n=252)^1^ | 6.47(0.15) | 7.29(0.19) | **0.12(0.07,0.17)**^3^ | **<0.001** |
|  | Model 2 (n=241)^2^ | 6.71(0.18) | 6.96(0.22) | 0.04(-0.04,0.12)^3^ | 0.352 |

Abbreviations: B, unstandardized beta coefficient; SE, standard error; CI, confidence interval; LPA, light-intensity physical activity; MVPA, moderate-to vigorous-intensity physical activity.

**Bold font** indicates p<0.05

^1^ In model 1, the clustering effect of childcare centers and the repeated effect of time were included as random intercepts, and time and group were included as fixed effect.

^2^ In model 2, the clustering effect of childcare centers and the repeated effect of time were included as random intercepts, and time and covariates (group, child age, child race/ethnicity, parental education, mean temperature, mean precipitation) were included as fixed effects. Mean temperature was added to model 2 because it was significantly different between baseline and follow-up in the accreditation and control groups.

^3^ Given the non-normal distribution of data for MVPA, the unstandardized beta coefficient was calculated for Log (MVPA).

**Supplementary Table 2.** Stratified analyses by group (accreditation, control) for the change in children’s BMI z-scores, expressive vocabulary and working memory (follow-up vs. baseline).

| Outcome variables | Groups | Baseline | Follow-up | Time | |
| --- | --- | --- | --- | --- | --- |
| **Model 1** | | | | | |
|  |  | Estimated marginal means (SE) | | B(95%CI) | P value |
| BMI z-score | Accreditation (n=121) | 0.56(0.09) | 0.63(0.10) | 0.07(-0.07,0.20) | 0.322 |
|  | Control (n=122) | 0.85(0.09) | 0.73(0.09) | **-0.13(-0.21,-0.04)** | **0.005** |
| Expressive vocabulary^1^ | Accreditation (n=67) | 18.01(1.10) | 22.15(1.15) | **4.14(3.14,5.14)** | **<0.001** |
|  | Control (n=46) | 20.24(0.99) | 26.22(0.91) | **5.98(4.72,7.23)** | **<0.001** |
|  |  | Estimated marginal means (SE) | | OR (95%CI)^2^ | P value |
| Working memory^1^ | Accreditation (n=67) | 0.29(0.06) | 0.67(0.06) | **5.03(2.26,11.20)** | **<0.001** |
|  | Control (n=46) | 0.64(0.09) | 0.69(0.09) | 1.33(0.57,3.10) | 0.506 |
| **Model 2** | | | | | |
|  |  | Estimated marginal means (SE) | | B(95%CI) | P value |
| BMI z-score | Accreditation (n=115) | 0.49(0.10) | 0.68(0.11) | **0.19(0.03,0.35)** | **0.023** |
|  | Control (n=118) | 0.80(0.10) | 0.86(0.10) | 0.07(-0.10,0.23) | 0.423 |
| Expressive vocabulary^1^ | Accreditation (n=63) | 20.91(1.00) | 20.56(1.07) | -0.03(-2.17,1.47) | 0.705 |
|  | Control (n=45) | 20.62(1.17) | 23.79(1.27) | **3.18(0.05,6.30)** | **0.047** |
|  |  | Estimated marginal means (SE) | | OR (95%CI)^2^ | P value |
| Working memory^1^ | Accreditation (n=63) | 0.35(0.07) | 0.63(0.08) | **3.24(1.32,7.97)** | **0.011** |
|  | Control (n=45) | 0.70(0.12) | 0.50(0.15) | 0.44(0.12,1.54) | 0.196 |

Abbreviations: SE, standard error, CI; confidence interval; BMI; body mass index.

**Bold font** indicates p<0.05.

In model 1, the clustering effect of childcare centers and the repeated effect of time were included as random intercepts, and time and group were included as fixed effects.

In model 2, the clustering effect of childcare centers and the repeated effect of time were included as random intercepts, and time, group, and covariates (child age, child race/ethnicity, parental education, and mean precipitation) were included as fixed effects.

^1^ Vocabulary (ranging from 0-45) and working memory (value “0” represents a score ≤1 [range: 0.00-1.00]; value “1” represents a score >1 [range: 1.33-3.67]) were measured in preschool children only.

^2^ Estimate for time (follow-up vs. baseline) can be interpreted as the odds of having better working memory at follow-up compared to baseline.
